# Hydrothorax

**Published:** 1852-08

**Authors:** Ira Manley

**Affiliations:** Markesan, Wis.


THE NORTH-WESTERN

MEDICAL AND SURGICAL JOURNAL.

NEW SERIES.

VOL. I. AUGUST, 1852. No. 4.

### ORIGINAL COMMUNICATIONS.

ARTICLE I.—*Hydrothorax.* By IRA MANLEY, M.D.

IT is well known that a great embarrassment, in the treatment of this disease, often arises from the uncertainty of tests in arriving at a positive knowledge of the condition of the lungs. If a lung is stuffed with tubercle, and a consequent touch of pleurisy has given rise to the effusion, it might be of importance to know that such is the case, that the treatment may be directed to correct the strumous diathesis, as well at the same time to produce absorption. If, on the other hand, the disease is a result of organic change in the heart or liver, or pure pleurisy, or if causes which are inappreciable have given rise to it, such treatment might be superfluous. Mr. Watson says, "We shall find two sets of symptoms to be distinguished, viz., those which depend on the primary disease, and those which depend on the collected fluid.

Doubtless, in an early stage of hydrothorax from tubercle, the physical signs would give notice of their presence; but as softening may not take place and the lung become collapsed, how are we to determine on the presence or absence of tubercle?

Aug. 6, 1850. Was called to see Mr. H———, æt. 48,—had been sick about a year—attended by a physician of extensive practice. Patient said he had black jaundice—complained of bowels, which were tympanitic—said his lungs were quite well.

Examined chest and found left side perfectly dull, intercostal spaces bulging, diameter exceeding right side by one inch, heart entirely to the right of sternum,—cough very slight, breathing not affected. Told patient that the side was filled with fluid; after thinking of it a little, he said he had at times called that side his "turtle shell" from the sensation of stiffness.

The tympanitis soon became very urgent and dyspnœa was a result,—as a last resort, after consultation, drew off five pints of a reddish fluid by paracentesis.

Rallied a few days, but finally sank.

Autopsy. Raised the sternum and dipped nearly one half pail of fluid from left side,—lung bound down to its origin in less compass than the closed hand would occupy, stuffed with tubercle,—heart bound fast entirely to right of sternum, healthy,—right lung healthy,—did not open cavity of the abdomen. Can the presence of tubercle be discovered before death in such a case?

April 1, 1851. Was called to see G. W., æt. 38,—rather plethoric. From the expression of the face I suspected disease of the heart,—complains of dyspnœa,—diagnosed hydrothorax of right side. The family is phthisical. I treated the case as is common, without reference to the strumous condition which existed. After three weeks, consulted with the gentleman who had treated the former case; he diagnosed "Milli's Asthma." As my treatment was useless except to make the patient comfortable, I was quite willing to resign it. No benefit resulted from his treatment, and two subsequent consultations with other gentlemen confirmed my opinion.

General anasarca followed,—the chest filled up, the patient's legs burst, and the last time I saw him the water was running across the floor from them.

3d Case. Female, æt. 31,—strumous habit; fluid occupied left side, to the entire exclusion of respiration, below the space between second and third ribs. From the unfortunate result of the former cases I varied the treatment.

Externally used tinc. iodine freely, and to soften the cuticle, bathed it with cod liver oil; applied a succession of blisters, by means of strong aqua ammonia, a little to the left of the spine. Internally, hyd. potash 9 grs. each day; cod liver oil ℥i three times a day for two weeks, patient disliked it so much discontinued it; gave some sweet spt. nitre and Prof. Mettaner's aperient solution (Braithwaite's Retrospect, part 23, p. 300,) for the bowels,—muriated tinc. opium, to quiet irritation.

The last time I saw patient the breathing was going on very well as low as the 5th rib, she was employed with light work, and soon went East.

I have at present a fourth case, a man; the family had nearly all died of consumption in Maine. Now, after three weeks' treatment, similar to case 3d, his general health is improved but the physical signs are no better; above the third rib the sound is very resonant, the respiration is jerking. Shall I infer the presence of tubercle from this sound? Does the fluid contained in the cavity below the third rib modify these sounds? If not, I may infer the presence of tubercle.

In all the cases of this disease I have seen, I have inferred the presence of tubercle, but it has been inference without positive proof; in a case of pure phthisis we suppose we have positive proof of the condition of the lung, but with the accumulation of fluid these proofs, to me, become uncertain; and to commence what will commonly be a long course of medical prescribing, without a tolerably certain knowledge of the cause of the difficulty, is anything but pleasant. We treat the effusion as a disease *per se*, after having diagnosed its presence. If we would enquire for its cause, and pay more attention to that, perhaps the reproach, that chronic hydrothorax is almost invariably a fatal disease, might be made with less justice.

MARKESAN, WIS., June 30, 1852.

